# Role and molecular mechanism of traditional Chinese medicine in preventing cardiotoxicity associated with chemoradiotherapy

**DOI:** 10.3389/fcvm.2022.1047700

**Published:** 2022-11-07

**Authors:** Xin-Fang Lv, Ruo-Qing Wen, Kai Liu, Xin-Ke Zhao, Chen-Liang Pan, Xiang Gao, Xue Wu, Xiao-Dong Zhi, Chun-Zhen Ren, Qi-Lin Chen, Wei-Jie Lu, Ting-Yan Bai, Ying-Dong Li

**Affiliations:** ^1^School of Traditional Chinese and Western Medicine, Gansu University of Chinese Medicine, Lanzhou, China; ^2^Key Laboratory of Prevention and Treatment for Chronic Diseases by Traditional Chinese Medicine, University Hospital of Gansu Traditional Chinese Medicine, Lanzhou, China; ^3^Affiliated Hospital of Gansu University of Chinese Medicine, Lanzhou, China; ^4^The First Hospital of Lanzhou University, Lanzhou, China; ^5^Lanzhou University Second Hospital, Lanzhou, China

**Keywords:** cardio-oncology, cardiotoxicity, chemoradiotherapy, traditional Chinese medicine, pharmacology, alternative treatments

## Abstract

Cardiotoxicity is a serious complication of cancer therapy. It is the second leading cause of morbidity and mortality in cancer survivors and is associated with a variety of factors, including oxidative stress, inflammation, apoptosis, autophagy, endoplasmic reticulum stress, and abnormal myocardial energy metabolism. A number of studies have shown that traditional Chinese medicine (TCM) can mitigate chemoradiotherapy-associated cardiotoxicity *via* these pathways. Therefore, this study reviews the effects and molecular mechanisms of TCM on chemoradiotherapy-related cardiotoxicity. In this study, we searched PubMed for basic studies on the anti-cardiotoxicity of TCM in the past 5 years and summarized their results. *Angelica Sinensis, Astragalus membranaceus* Bunge, Danshinone IIA sulfonate sodium (STS), Astragaloside (AS), Resveratrol, Ginsenoside, Quercetin, Danggui Buxue Decoction (DBD), Shengxian decoction (SXT), Compound Danshen Dripping Pill (CDDP), Qishen Huanwu Capsule (QSHWC), *Angelica Sinensis* and *Astragalus membranaceus* Bunge Ultrafiltration Extract (AS-AM),Shenmai injection (SMI), Xinmailong (XML), and nearly 60 other herbs, herbal monomers, herbal soups and herbal compound preparations were found to be effective as complementary or alternative treatments. These preparations reduced chemoradiotherapy-induced cardiotoxicity through various pathways such as anti-oxidative stress, anti-inflammation, alleviating endoplasmic reticulum stress, regulation of apoptosis and autophagy, and improvement of myocardial energy metabolism. However, few clinical trials have been conducted on these therapies, and these trials can provide stronger evidence-based support for TCM.

## Introduction

Chemoradiotherapy has improved survival in patients with cancer; however, the resulting cardiotoxicity is a major cause of morbidity and mortality in the oncology population (1–3). In the United States, women have a significantly increased risk of death from cardiotoxicity, which exceeds the risk of death from cancer or recurrence, and it is the leading cause of death in patients over 50 years old with breast cancer (4). The cumulative incidence of chronic heart failure (CHF) 10 years after systemic therapy in Dutch patients with early-stage breast cancer was 4.8% (5). Anthracycline use in first-line lymphoma treatment is associated with a significantly increased incidence of CHF in Danish patients with lymphoma (6). The incidence of childhood cancer cardiotoxicity after anthracycline therapy in a multi-ethnic Asian population was 7%, of which 37.5% had CHF (7). Radiotherapy also causes cardiotoxicity, which has a 4–16% relative risk of heart disease and major cardiac events per Gray of the average cardiac radiation dose (8). Radiotherapy induces oxidative stress (OS) and matrix remodeling, which alters the cardiac microvascular and macrovascular environment and induces coronary artery disease, myocardial fibrosis, and cardiomyopathy, valvular disease, pericardial disease, and arrhythmias (9, 10); chemotherapies can cause cardiotoxicity through OS, lipid peroxidation, and inhibition of topoisomerase IIβ (Top2β), leading to cardiomyocytes (CMs) damage (11).

Cardiac oncology clinical practice guidelines define cardiotoxicity as (1) a relative decrease in overall longitudinal echocardiographic strain of >15% or a new increase in cardiac biomarkers in individuals with left ventricular ejection fraction (LVEF) ≥50%, (2) a decrease in LVEF to 40–49% (accompanied by a relative decrease in overall longitudinal echocardiographic strain of >15% or a decrease in LVEF of <10% and a new increase in new cardiac biomarkers), or (3) a decrease in LVEF to <40% (12). However, chemoradiotherapy not only affects resting LVEF but also has a wide range of effects on the entire cardiovascular system, including direct effects on cardiac structure (e.g., fibrosis), diastolic function, cardiac conduction and arrhythmias, systemic and pulmonary vascular function and hemodynamics, hemostasis and thrombosis, and cardiac response to injury and stress (13). Statins, angiotensin-converting enzyme inhibitors, angiotensin receptor blockers, beta-blockers, and dexrazoxane are currently used clinically to prevent or reduce radiotherapy-related cardiotoxicity (14–21). However, these drugs do not significantly reduce the risk of cardiotoxicity; instead, they interfere with the antitumor properties and prognostic benefits of anthracyclines, increasing the incidence of secondary malignancies (11, 14, 18). Therefore, there is an urgent need to explore safer and more effective options.

TCM has been used in China for thousands of years to treat human diseases and has attracted widespread attention from other countries owing to its unique healing properties (22). In recent years, TCM has made contributions to global public health, such as artemisinin for treating malaria (23), arsenic trioxide for acute promyelocytic leukemia (24, 25), and played an important role in treating pneumonia associated with novel coronavirus disease 2019 (COVID-19) (26–30). In 2019, the World Health Organization included TCM as an accepted form of treatment in its International Statistical Classification of Diseases (ICD-11) for the first time in the 72nd World Health Assembly, reflecting the contribution of TCM to global healthcare (31). Cancer and cardiovascular diseases are two major maladies that pose a serious threat to human health, and cardiovascular toxicity caused by these cancer treatments poses a serious and specific threat to the health and survival of patients with cancer. Recent studies have shown that TCM can combat chemoradiotherapy-related cardiotoxicity without affecting the antitumor activity of the treatment (32). However, the mechanism of action of TCM for the treatment of chemoradiotherapy-related cardiotoxicity is not fully understood. Therefore, we summarize recent studies on the prevention and treatment of chemoradiotherapy-related cardiotoxicity by TCM and explain its mechanism to provide a basis for the prevention and treatment of chemoradiotherapy-related cardiotoxicity by TCM.

## Molecular mechanisms of chemoradiotherapy-related cardiotoxicity and the therapeutic effects of traditional Chinese medicine

**Figure 1:**
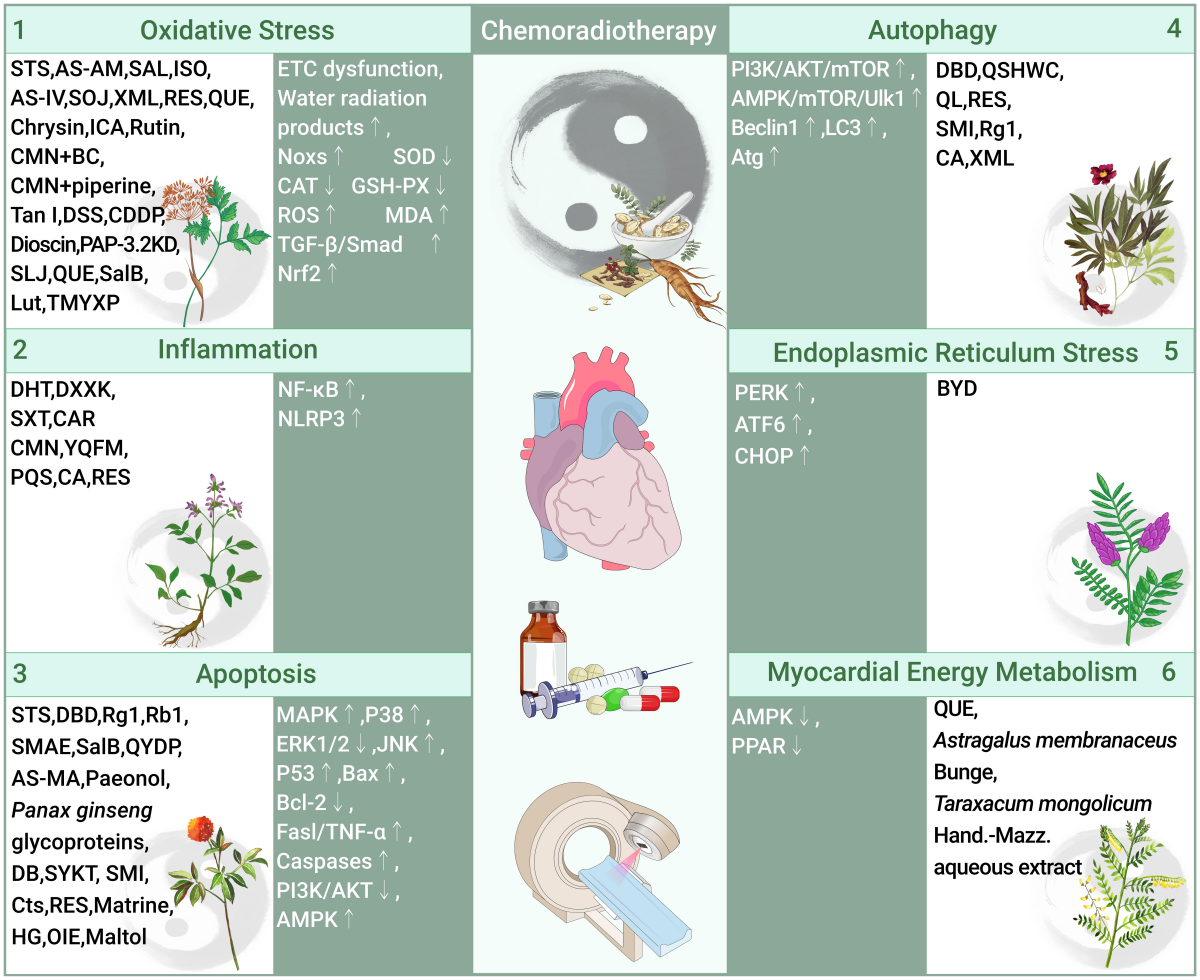
Role and molecular mechanism of traditional Chinese medicine in preventing cardiotoxicity associated with chemoradiotherapy (traditional Chinese medicine alleviates chemoradiotherapy-related cardiotoxicity by inhibiting oxidative stress, anti-inflammatory, regulating apoptosis and autophagy, inhibition of endoplasmic reticulum stress, and improves myocardial energy metabolism. OS, Oxidative stress; Noxs, NADPH oxidases; SOD, Superoxide dismutase; CAT, Catalase; GSH-PX, Glutathione; ROS, Reactive oxygen species; MDA, Malondialdehyde; TGF-β, Transforming growth factor-β; Smad, Small Mothers Against Decapentaplegic; Nrf2, Nuclear factor erythroid 2-related factor 2; STS, Tanshinone IIa sodium sulfonate; AS-AM, *Angelica Sinensis* and *Astragalus membranaceus* Bunge Ultrafiltration Extract; SAL, Salidroside; ISO, Isoorientin; AS-IV, Astragaloside IV; SOJ, Steroidal saponins extract from *Ophiopogon japonicus* (SOJ) root; XML, Xinmailong injection; RES, Resveratrol; QUE, Quercetin; ICA, Icariin; CMN, Curcumin; BC, β-carotene; Tan I, Tanshinone I; DSS, Danshensu; CDDP, Compound Danshen Dripping Pill; PAP-3.2KD, Pilose antler Peptide-3.2KD; SLJ, Shenlijia; SalB, Salvianolic acid B; Lut, Luteolin; TMYXP, Tongmai Yangxin Pills; NF-κB, Nuclear factor kappa-B; NLRP3, Sirtuin1 (Sirt1)-nod-like receptor protein 3; DHT, Dihydrotanshinone I; DXXK, Di'ao Xinxuekang capsule; SXT, Shengxian decoction; CAR, Cardamom; YQFM, Yiqi Fumai lyophilized injection; PQS, *Panax quinquefolius*; CA, Calycosin; RES, Resveratrol; MAPK, Mitogen-activated protein kinase; ERK, Extracellular signal-regulated kinases; JNK, C-Jun-terminal kinases; Bax, Bcl-2 associated X; Bcl-2, B-cell lymphoma-2; Fasl/TNF-α, Fas ligand/tumor necrosis factor-α; Caspases, Cysteine aspartate proteases; PI3K/AKT, phosphatidylinositol 3-kinase/serine-threonine protein kinase; AMPK, adenosine monophosphate-activated protein kinase; DBD, Danggui Buxue decoction; Rg1, ginsenoside Rg1; Rb1, ginsenoside Rb1; SMAE, *Salvia miltiorrhiza* aqueous extract; QYDP, Qishen Yiqi Dropping Pills; DB, Diethyl blechnic; SYKT, Sanyang Xuedai; SMI, Shenmai Injection; Cts, Cryptotanshinone; HG, Higenamine; OIE, Oroxylum; mTOR, Mechanistic Target Of Rapamycin; LC3, Light chain-3; Atg, Autophagy-related genes; QSHWC, Qishen Huanwu Capsule; QL, Qiliqiangxin; PERK, The stress protein kinase R-like endoplasmic reticulum kinase; ATF6, Activating transcription factor 6; CHOP:C/EBP homologous protein; BYD, Baoyuan decoction; PPAR, peroxisome proliferator-activated receptors).

The pathogenesis of chemoradiotherapy cardiotoxicity is associated with multiple molecular pathways, with OS and inflammation being the most important pathways, along with apoptosis, autophagy, endoplasmic reticulum stress, and abnormal myocardial energy metabolism (Table 1, Figure 1).

**Table 1:** Protective effects and mechanisms of traditional Chinese medicine against chemoradiotherapy-related cardiotoxicity.

**Function**	**Radiotherapy/** **Chemotherapy**	**Traditional** **Chinese medicine**	**Molecular mechanisms**	**Research type**	**References**
Anti-oxidative Stress	Radiotherapy	STS	ROS and MDA reduction, Increase SOD	*in vitro*	(48, 49)
		AS-AM	Inhibition of TGF-β/Smad	*in vivo, in vitro*	(51–53)
	Chemotherapy (Anthracyclines)	SAL	ROS reduction	*in vivo, in vitro*	(57, 58)
		ISO	ROS reduction	*in vitro*	(59)
		AS-IV	Reduction of Nox2, Nox4	*in vivo, in vitro*	(61)
		Tan I	activates Nrf2	*in vivo, in vitro*	(65)
		DSS	regulating Keap1-Nrf2/NQO1	*in vivo*	(66)
		CDDP	Reduces ROS, MDA, activates Nrf2	*in vivo*	(67)
		Dioscin	Regulates ROS, activation of Nrf2	*in vivo*	(68)
		SOJ	Increases SOD, CAT, GSH-Px, decreases MDA, and inhibits OS	*in vivo*	(70)
		XML	Increases MDA, SOD	*in vitro*	(71)
		RES	ROS reduction	*in vitro*	(72)
		Crocin	ROS MDA and TOS reduction, increase TAC	*in vitro*	(73)
		PAP-3.2KD	Inhibition of TGF-β/Smad	*in vivo*	(75)
		SLJ	Upregulates TIMP-1/2/3	*in vivo*	(76)
	Chemotherapy (non-anthracycline)	QUE	Reduces ROS levels	*in vivo, in vitro*	(78)
		Chrysin	Increases SOD, CAT, and GSH, decreases MDA	*in vivo*	(79)
		CMN+BC	Decreases MDA, increases CAT and SOD	*in vivo*	(80)
		CMN+piperine	Increases SOD and CAT	*in vivo*	(81, 82)
		ICA	Regulates GSH-Px, CAT, SOD, and MDA	*in vivo, in vitro*	(83)
		Rutin	Increases MDA blocking and decreases tGSH levels	*in vivo*	(84)
		QUE	Regulates Nrf2	*in vitro*	(85)
		SalB	Regulates Nrf2	*in vitro*	(86, 87)
		Lut	Regulates Nrf2	*in vitro*	(88)
		TMYXP	Regulates Nrf2/HO-1, p38 MAPK	*in vivo, in vitro*	(89)
Anti-inflammatory	Chemotherapy	DHT	Decreases NF-κB	*in vivo, in vitro*	(92)
		DXXK	Reduces ROS levels; downregulates NF-κB p65	*in vivo, in vitro*	(93)
		SXT	Inhibit NF-κB	*in vitro*	(94)
		CAR	Decreased NF-κB	*in vivo*	(95)
		CMN	Decreased NF-κB	*in vivo*	(96)
		YQFM	Decreased NF-κB	*in vivo*	(97)
		PQS	NF-κB inhibition and regulation of PI3K/Akt	*in vivo*	(98)
		CA	Inhibit NLRP3	*in vivo, in vitro*	(99)
		RES	Inhibit NLRP3	*in vivo*	(100)
Reduced apoptosis	Radiotherapy	STS	Downregulates P38,caspase-3, upregulates P ERK1/2 and Bax	*in vitro*	(49)
		DBD	Reduces Fasl/TNF-α	*in vivo*	(105)
	Chemotherapy (anthracyclines)	Ginsenoside Rg1	Increases in Akt, Erk, Bcl-2/Bax. Decreases Cyt-c	*in vivo*	(106)
		Ginsenoside Rb1	Decreases caspase-3 and caspase-8	*in vitro*	(107)
		SMAE	Modulated ERK/p53/Bcl-xL/caspase-3	*in vitro*	(108)
		SalB	Promoted Bcl-2	*in vivo*	(109)
		QYDP	Upregulates Bax, downregulates Bcl-2	*in vivo*	(110)
		AS-AM	Downregulates Bax, Caspase-3, Caspase-12, and upregulates Bcl-2	*in vitro*	(111–113)
		Paeonol	Upregulated Bcl-2 and mitochondrial Cyt c, downregulated Bax, caspase-3, and cytoplasmic-Cytc	*in vivo*	(114)
		*Panax ginseng* glycoproteins	Regulates MAPK	*in vitro*	(116)
		DB	JNK1/2	*in vitro*	(117)
		SYKT	Inhibits p53, MAPK	*in vitro*	(118, 119)
		SMI	Decreases Bax/Bcl-2 and Caspase-3 levels; increases PI3K, p-Akt, p-GSK-3β, AMPK	*in vivo, in vitro*	(120, 123)
		Cts	Regulation Akt-GSK-3β-mPTP	*in vitro*	(121)
		RES	Regulates AMPK	*in vitro*	(124)
		Matrine	Regulates AMPK	*in vivo, in vitro*	(125)
		Higenamine	ROS reduction, AMPK inhibition	*in vivo, in vitro*	(126)
	Chemotherapy (non-anthracycline)	OIE	ROS inhibition	*in vivo*	(127)
		Maltol	ROS reduction	*in vitro*	(128)
Regulation of autophagy	Chemotherapy (anthracyclines)	DBD	Activates PI3K	*in vivo*	(134)
		QSHWC	Regulates PI3K/Akt, MAPK, MAPK8, FOXO, LC3	*in vivo, in vitro*	(135, 136)
		QL	Regulates PI3K/AKT/mTOR	*in vivo*	(137)
		RES	Regulates AMPK/mTOR/Ulk1	*in vitro*	(138)
		SMI	Regulates miR-30a/Beclin1, JNK	*in vivo, in vitro*	(142, 143)
		Ginsenoside Rg1	Downregulates LC3, Atg5, JNK 1, Beclin 1	*in vivo*	(144)
		CA	Atg7	*in vivo*	(145)
		XML	Downregulates Beclin 1, Atg7, P38, Erk1/2; upregulates PKB/Akt, PI3K, Bcl-2	*in vivo*	(146)
Inhibition of endoplasmic reticulum stress	Chemotherapy (anthracyclines)	BYD	Reduces GRP78, PERK, eIF2α, CHOP	*in vivo*	(149)
Improves myocardial energy metabolism	Chemotherapy (Anthracyclines)	QUE *Astragalus membranaceus* Bunge	Regulates AMPK, PPARα PPARγ	*in vivo, in vitro* *in vitro*	(152) (153)
		*Taraxacum mongolicum* Hand.-Mazz. aqueous extract	Activates P-gp	*in vivo, in vitro*	(154)

STS, Tanshinone IIa sodium sulfonate; AS-AM, *Angelica Sinensis* and *Astragalus membranaceus* Bunge Ultrafiltration Extract; SAL, Salidroside; ISO, Isoorientin; AS-IV, Astragaloside IV; Tan I, Tanshinone I; DSS, Danshensu; CDDP, Compound Danshen Dripping Pill; SOJ, Steroidal saponins extract from *Ophiopogon japonicus* (Thunb.) Ker Gawl root; XML, Xinmailong injection; RES, Resveratrol; PAP-3.2KD, Pilose antler Peptide-3.2KD; SLJ, Shenlijia; QUE, Quercetin; CMN, Curcumin; BC, β-carotene; ICA, Icariin; SalB, Salvianolic acid B; Lut, Luteolin; TMYXP, Tongmai Yangxin Pills; DHT, Dihydrotanshinone I; DXXK, Di'ao Xinxuekang capsule; SXT, Shengxian decoction; CAR, Cardamom; YQFM, Yiqi Fumai lyophilized injection; PQS, *Panax quinquefolius*; CA, Calycosin; DBD, Danggui Buxue decoction; SMAE, *Salvia miltiorrhiza* aqueous extract; QYDP, Qishen Yiqi Dropping Pills; DB, Diethyl blechnic; SYKT, Sanyang Xuedai; SMI, Shenmai Injection; Cts, Cryptotanshinone; OIE, Oroxylum; QSHWC, Qishen Huanwu Capsule; QL, Qiliqiangxin; BYD, Baoyuan decoction; ROS, Reactive oxygen species; MDA, Malondialdehyde; SOD, Superoxide dismutase; TGF-β, Transforming growth factor-β; Smad, Small Mothers Against Decapentaplegic; Noxs, NADPH oxidases; Nrf2, Nuclear factor erythroid 2-related factor 2; Keap-1, Recombinant Kelch Like ECH Associated Protein 1; HO-1, Heme Oxygenase 1; CAT, Catalase; GSH-PX, Glutathione peroxidase; OS, Oxidative stress; TOS, Total oxidant status; TAC, Total antioxidant capacity; TIMP, Matrix-metalloproteinase inhibitor; MAPK, Mitogen-activated protein kinase; NF-κB, Nuclear factor kappa-B; PI3K/AKT, phosphatidylinositol 3-kinase/serine-threonine protein kinase; NLRP3, Sirtuin1 (Sirt1)-nod-like receptor protein 3; Caspases, Cysteine aspartate proteases; ERK1/2, Extracellular signal-regulated kinases1/2; Bax, Bcl-2 associated X; Bcl-2, B-cell lymphoma-2; Fasl/TNF-α, Fas ligand/tumor necrosis factor-α; Cyt-c, Cytochrome C; JNKs, C-Jun-terminal kinases; GSK-3β, Glycogen synthase kinase 3 beta; AMPK, Adenosine monophosphate-activated protein kinase; FOXO, Forkhead Box O; LC3, Light chain-3; mTOR, Mechanistic Target Of Rapamycin; ULK1, Unc-51-like autophagy activated kinase 1; Atg, Autophagy-related genes; miR, microRNAs; GRP78, Glucose regulated protein78; PERK, The stress protein kinase R-like endoplasmic reticulum kinase; eIF2α, Eukaryotic translation initiation factor 2-alpha; CHOP, C/EBP homologous protein; PPAR, Peroxisome proliferator-activated receptors; P-gp, P-glycoprotein.

### Oxidative stress

OS refers to the imbalance of pro-oxidants and antioxidants and the disruption of redox signaling and control (33). The mitochondrial respiratory chain and nicotinamide adenine dinucleotide phosphate (NADPH) are the main cellular sources of reactive oxygen species (ROS) (34). NADPH oxidases (Noxs) are a group of plasma membrane-associated enzymes that are among the most important sources of ROS, Nox2 and Nox4 are the major cardiac isoforms (35), overexpression of Nox2 and Nox4 induces the production of ROS (36). Nuclear factor erythroid 2-related factor 2 (Nrf2) controls gene expression of endogenous antioxidant synthesis and ROS-eliminating enzymes in response to various electrophilic compounds, inactivates the negative regulator Kelch-like ECH-associated protein 1 (Keap1), and activates Nrf2 by overexpression of mitochondrial ROS (mtROS) and Nox2 and Nox4 (37). The role of antioxidant enzyme systems [superoxide dismutases (SODs), catalases (CAT), glutathione peroxidases (GPxs), and paraoxonases (PONs)] is to scavenge ROS (38), and oxidative damage occurs when ROS production exceeds the buffering capacity of ROS scavengers or when the antioxidant defense system is defective (39). Increased ROS also caused the development of myocardial fibrosis (MF) (40). Transforming growth factor-β (TGF-β) is a key factor in MF, and ROS is an immediate activator of TGF-β1.

#### Radiotherapy-induced oxidative stress

Oxidative stress and ROS may be the main cause of ionizing radiation (IR)-induced cardiotoxicity (41). IR leads to mitochondrial electron transport chain (ETC) dysfunction and ROS overproduction, causing DNA damage and protein and lipid peroxidation, the latter of which leads to the production of malondialdehyde (MDA) (39, 41–43); IR activates Noxs and inhibits SOD expression for ROS production and accumulation (44). Tissues and cells, which are 80% water, rapidly undergo OS responses after being targeted by ionizing radiation (45), forming water radiolysis products rich in ROS and releasing ROS (46). The late effects of radiotherapy are due to IR and IR-induced production of chronic free radicals from water molecules in the surrounding environment (47). STS significantly inhibits the increase of ROS and MDA content in H9C2 cells and cardiac fibroblasts (CFS) under X-ray radiation and increased the level of SOD (48, 49).

**Figure 2:**
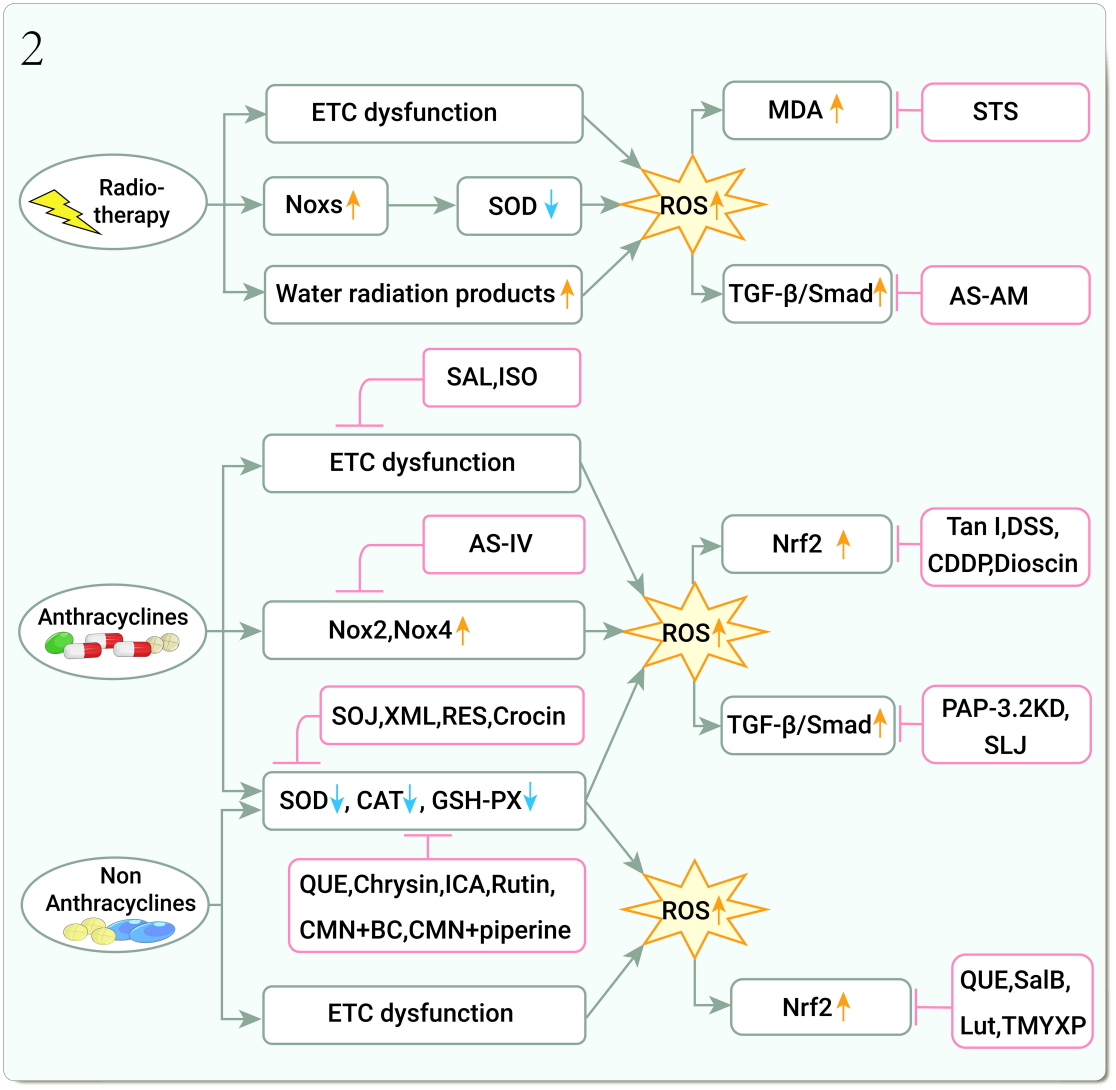
Traditional Chinese medicine alleviates cardiotoxicity associated with chemoradiotherapy by inhibiting oxidative stress (Noxs, NADPH oxidases; SOD, Superoxide dismutase; CAT, Catalase; GSH-PX, Glutathione peroxidase; ROS, Reactive oxygen species; MDA, Malondialdehyde; TGF-β, Transforming growth factor-β; Smad, Small Mothers Against Decapentaplegic; Nrf2, Nuclear factor erythroid 2-related factor 2; STS, Tanshinone IIa sodium sulfonate; AS-AM, *Angelica Sinensis* and *Astragalus membranaceus* Bunge Ultrafiltration Extract; SAL, Salidroside; ISO, Isoorientin; AS-IV, Astragaloside IV; SOJ, Steroidal saponins extract from *Ophiopogon japonicus* (SOJ) root; XML, Xinmailong injection; RES, Resveratrol; QUE, Quercetin; ICA, Icariin; CMN, Curcumin; BC, β-carotene; Tan I, Tanshinone I; DSS, Danshensu; CDDP, Compound Danshen Dripping Pill; PAP-3.2KD, Pilose antler Peptide-3.2KD; SLJ, Shenlijia; SalB, Salvianolic acid B; Lut, Luteolin; TMYXP, Tongmai Yangxin Pills).

Myocardial fibrosis is a late manifestation of radiation-induced heart disease (RIHD) (50). The current studies showed that AS-AM, a DBD-derivative, downregulated TGF-β/Smad and COL-I expression in an X-ray-induced rat CFs fibrotic injury model (51–53) (Figure 2).

#### Oxidative stress induced by anthracycline-based chemotherapeutic agents

Doxorubicin (DOX)-induced OS is thought to be a major cause of cardiotoxicity (54, 55). DOX alters myocardial ETC gene expression and translation *in vivo*, reducing the redox cycle of the ETC complex I, and generates large amounts of ROS (56). Disrupting this process, the salidroside (SAL) attenuates DOX-induced cardiac insufficiency by reducing ROS production and improving mitochondrial function (57, 58). Another TCM, isoorientin (3′,4′,5,7-tetrahydroxy-6-C-glucopyranosyl flavone) is a natural C-glycosyl flavonoid with strong free radical scavenging activity that reduces ROS, maintains mitochondrial function, and attenuates DOX-induced H9C2 CMs damage (59).

DOX induces Noxs activation, which leads to increased ROS production (60). The compound AS-IV attenuates DOX-induced Nox2 and Nox4 expression, OS, and cardiomyopathy in CMs (61). Nrf2 deficiency exacerbates DOX-induced cardiotoxicity and cardiac insufficiency (62–64). Playing a role in these pathways, Tanshinone I (Tan I) upregulated key proteins in the Nrf2 pathway to improve cardiac function and protect against both *in vivo* and *in vitro* DOX-induced myocardial structural damage in mice (65). Danshensu (DSS) effectively exerted anti-oxidative stress, anti-inflammatory, and anti-apoptotic therapeutic effects against DOX-induced cardiotoxicity by regulating the expression of Keap1-Nrf2/NQO1 (66). The CDDP activates Nrf2 expression to reduce the levels of ROS, MDA, and cardiac damage in mice (67). Dioscin, an extract from the rhizome of *Dioscorea punctata*, also inhibits myocardial oxidative damage by activating the Nrf2 pathway, lowers Keap1 expression, and attenuates cardiotoxicity (68).

DOX significantly reduces antioxidant enzyme levels, leading to redox imbalances and increased OS, but these effects can be treated with TCMs (69). Steroidal saponins extract from *Ophiopogon japonicus* (Thunb.) Ker Gawl root (SOJ) increased SOD, CAT, and GSH-Px activities and decreased MDA in rat myocardial tissue by inhibiting OS (70). In H9C2 cells, XML decrease DOX-induced MDA content, enhance SOD activity, increase ROS scavenging, and attenuate cardiotoxicity (71); RES reduced DOX-induced ROS content and improved cell survival, the effect of RES against DOX cardiotoxicity was comparable to that of dexrazoxane and carvedilol (72); additionally, crocin can reduce ROS, MDA and total oxidant status (TOS) levels, increase total antioxidant capacity (TAC), mitigation of DNA damage (73).

DOX both activated the TGF-β and P-Smad3 signaling pathways and enhanced collagen deposition in CMs (74), inducing MF development. Pilose antler peptide-3.2KD (PAP-3.2KD) has multiple biological activities in cardiomyopathy and reverses histological changes in cardiac tissue by decreasing TGF-β1, Smad2/3/4, and P-Smad2/3 levels, elevating Smad7 protein levels, thereby regulating pathological changes in the TGF-β/Smad signaling pathway, such as myofascial disorders, MF, and diffuse CMs edema (75). Shenlijia (SLJ) can improve cardiac function and inhibit MF progression. It improves cardiac function and ultrastructure, and inhibits MF development in DOX-induced CHF rats by upregulating extracellular matrix-metalloproteinase inhibitor (TIMP) expression (76) (Figure 2).

#### Oxidative stress induced by non-anthracycline chemotherapeutic agents

Aside from DOX, non-anthracycline chemotherapeutic agents, such as cyclophosphamide (CyC) and cisplatin (CP), cause cardiotoxicity by inducing mitochondrial dysfunction, leading to ETC damage, oxidative phosphorylation, decreased antioxidant enzyme levels and antioxidant capacity, increased ROS, activate Nrf2 and induce apoptosis (77).

CyC, an alkylating agent, combined with DOX is the most commonly used regimen for triple-negative breast cancer chemotherapy. CyC cardiotoxicity is caused by OS. Quercetin (QUE) has strong antioxidant activity and attenuates CyC-induced cardiotoxicity by inhibiting ROS accumulation in CMs; interestingly, it also enhances the antitumor activity of CyC (78). Another TCM that combats CyC-effects, chrysin increases enzyme levels that combat ROS and decreases levels of lipid peroxidation products in CyC-injured hearts, all of which protect against cardiotoxicity (79).

CP is another alkylating agent. Curcumin (CMN) has strong antioxidant effects, and when combined with β-carotene (BC), reduces lipid peroxidase product and increases anti-OS enzyme activities in CP-treated rat heart tissue (80). An additional CMN combination with piperine significantly increases anti-OS enzyme levels in the cardiac tissue of CP-treated rats (81, 82). Icariin (ICA) and rutin, attenuated CP-induced myocardial injury by increasing anti-OS enzyme and decreasing lipid peroxidase product levels (83, 84). QUE, salvianolic acid B (SalB), and luteolin (Lut) significantly reduced CP-induced OS by regulating Nrft2 signaling pathways (85–88). The 11-herbs combination treatment, Tongmai Yangxin Pills (TMYXP), nourish Qi and Yin, promoting blood circulation, relieving pain, and can improve the anti-OS ability of CP chemotherapy CMs by regulating Nrf2/HO-1 pathway and p38 MAPK pathway (89) (Figure 2).

OS is an important cause of disease, and this is no exception in cardiotoxicity due to chemoradiotherapy. Radiotherapy leads to increased ROS and MDA levels, decreased SOD levels, and upregulated TGF-β1 expression leading to MF, and STS and AS-AM can reverse these damages; chemotherapy leads to increased endogenous ROS production and decreased antioxidant enzyme expression, and the above-mentioned chemotherapy drugs counteract DOX-induced cardiotoxicity by reducing ROS production, inhibiting Nox2 and Nox4 overexpression, regulating Nrf2 function, and the above TCM counteracted DOX-induced cardiotoxicity by reducing ROS production, inhibiting Nox2 and Nox4 overexpression, regulating Nrf2 function, and increasing antioxidant enzyme content. However, there are few studies on the mechanism of radiotherapy-related cardiotoxicity in TCM, and there is a lack of studies on important factors and pathways such as Noxs and Nrf2. In the studies on chemotherapy-related cardiotoxicity, there are no studies on Top2β, which is a key factor leading to cardiotoxicity. In the future, more and more in-depth studies on key targets are needed.

### Inflammation

Inflammation is another important factor in chemotherapy drug-related cardiotoxicity and is related to OS. Nuclear factor kappa-B (NF-κB) is a key transcription factor in the inflammatory response, sirtuin1 (Sirt1)-nod-like receptor protein 3 (NLRP3) inflammatory vesicles are protein complexes that activate the secretion of the pro-inflammatory cytokine interleukin (IL)-1β in a cysteine aspartate proteases (caspases)-1-dependent manner and are involved in inflammatory regulation. DOX activates NF-κB and NLRP3 inflammatory vesicles, causing cardiotoxicity (90, 91).

Dihydrotanshinone I (DHT) upregulates transcription factor EB (TFEB) nuclear expression and decreases p-IKKα/β and p-NF-κB expression, and is used for anti-inflammatory management of DOX induced cardiotoxicity *via* the mammalian target of rapamycin (mTOR)-TFEB-NF-κB signaling pathway (92). The main component of Di'ao Xinxuekang capsule (DXXK) is diosgenin, protects against cardiotoxicity by reducing ROS and downregulating NF-κB p65 (93). Shengxian decoction (SXT) inhibits NF-κB activity, thus preventing cardiotoxicity from DOX treatment (94). Cardamom (CAR) decreased cardiac NF-κB levels ameliorating DOX-induced cardiotoxicity in rats (95). In DOX-induced cardiotoxicity in rats, CMN showed anti-inflammatory potential by reducing IFN-γ levels and immune expression of iNOS, NF-κB, and tumor necrosis factor-α(TNF-α) (96). Yiqi Fumai lyophilized injection (YQFM) pretreatment of DOX-intoxicated rats significantly inhibited the expression of NF-κB, TNF-α, and cyclooxygenase-2 (97). Saponins from the leaves of *Panax quinquefolius* (PQS) inhibit NF-κB activity and disrupt the phosphatidylinositol 3 kinase (PI3K) /protein kinaseB(AKT) (PI3K/Akt)apoptotic pathway, thus preventing cardiotoxicity from CP treatment (98).

**Figure 3:**
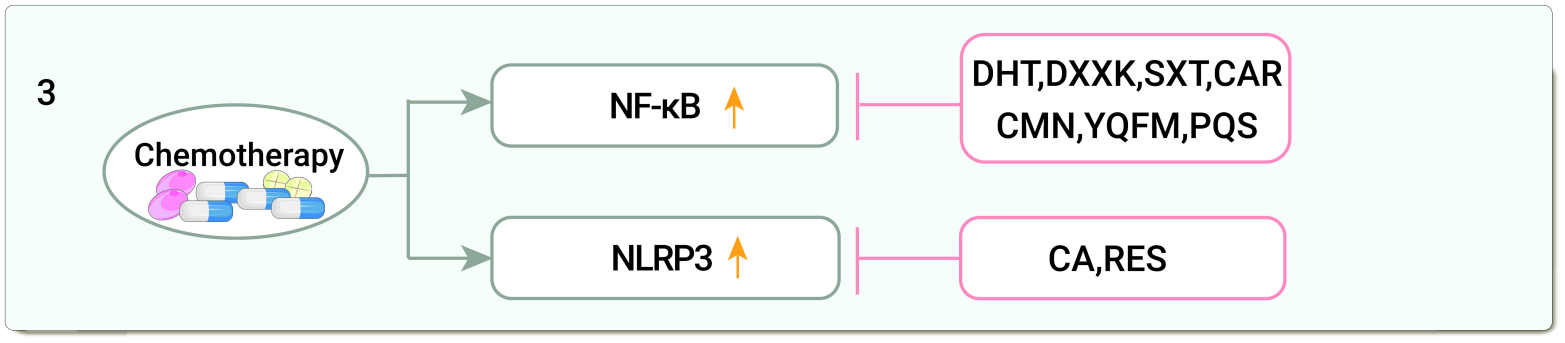
Traditional Chinese medicine alleviates cardiotoxicity associated with chemoradiotherapy by anti-inflammatory (NF-κB, Nuclear factor kappa-B; NLRP3, Sirtuin1 (Sirt1)-nod-like receptor protein 3; DHT, Dihydrotanshinone I; DXXK, Di'ao Xinxuekang capsule; SXT, Shengxian decoction; CAR, Cardamom; CMN, Curcumin; YQFM, Yiqi Fumai lyophilized injection; PQS, *Panax quinquefolius*; CA, Calycosin; RES, Resveratrol).

In DOX-treated cells and mouse hearts, levels of NLRP3 and related proteins were elevated, and calycosin (CA) ameliorated cardiotoxicity *via* the NLRP3 pathway (99). Resveratrol (RES) inhibition of NLRP3 inflammatory vesicle activation significantly reduced systemic inflammation and contributed to the improvement of DOX-induced myocardial injury and late-onset hypertension-induced cardiomyopathy in young mice (100) (Figure 3).

Among the inflammatory mechanisms, TCM for cardiotoxicity involves only two pathways, NF-κB and NLRP3, but other inflammatory pathways such as STAT1 and STAT3 also play important roles in the development of cardiotoxicity and should be of interest to investigators.

### Apoptosis

#### Radiotherapy-induced apoptosis

**Figure 4:**
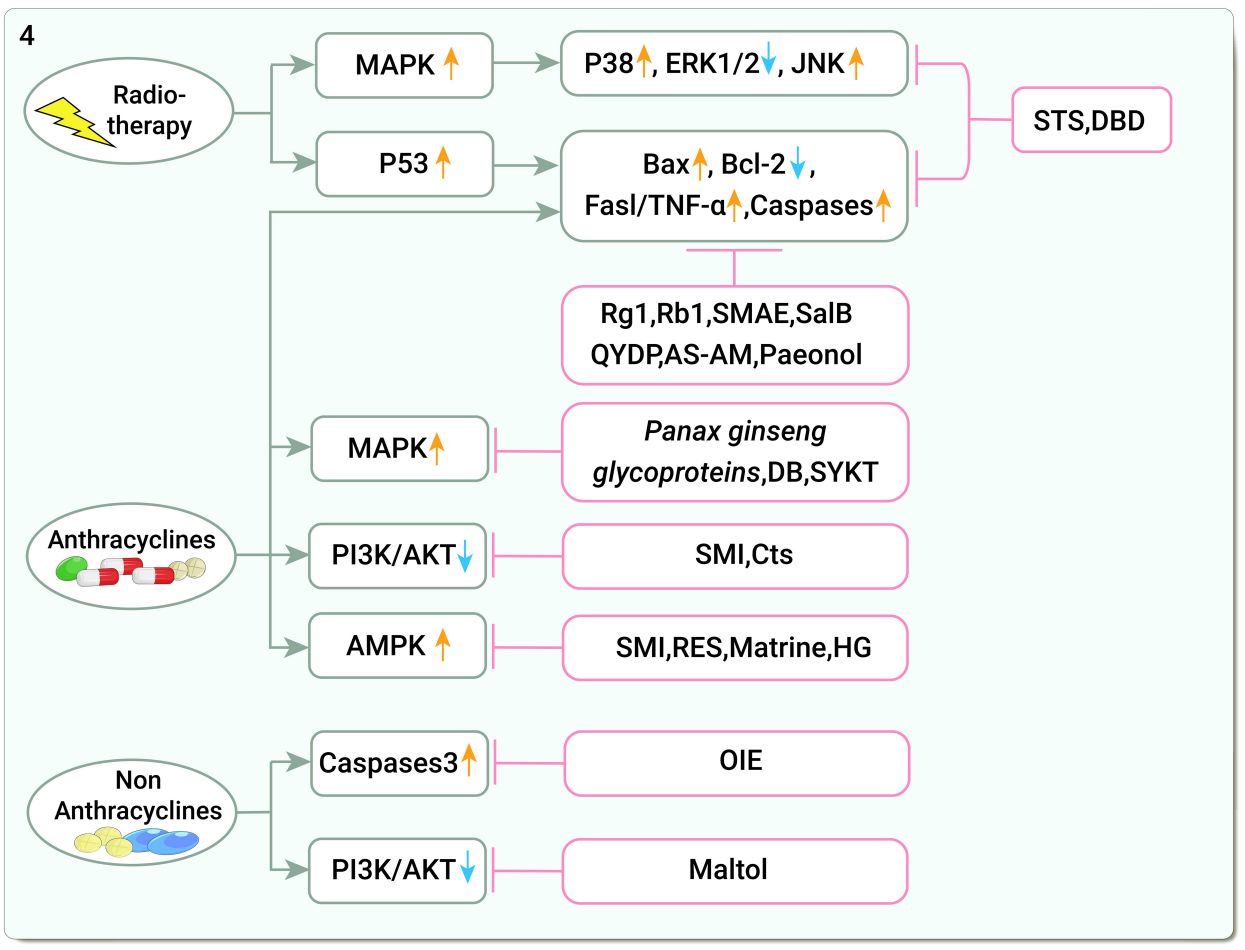
Traditional Chinese medicine alleviates cardiotoxicity associated with chemoradiotherapy by reducing apoptosis (MAPK, Mitogen-activated protein kinase; ERK1/2, Extracellular signal-regulated kinases1/2; JNKs, C-Jun-terminal kinases; Bax, Bcl-2 associated X; Bcl-2, B-cell lymphoma-2; Fasl/TNF-α, FasLigand/tumor necrosis factor-α; Caspases, Cysteine aspartate proteases; PI3K/AKT, Phosphatidylinositol 3-kinase/serine-threonine protein kinase; AMPK, Adenosine monophosphate-activated protein kinase; STS, Tanshinone IIa sodium sulfonate; DBD, Danggui Buxue decoction; Rg1, ginsenoside Rg1; Rb1, ginsenoside Rb1; SMAE, *Salvia miltiorrhiza* aqueous extract; SalB, Salvianolic acid B; QYDP, Qishen Yiqi Dropping Pills; AS-AM, *Angelica Sinensis* and *Astragalus membranaceus* Bunge Ultrafiltration Extract; DB, Diethyl blechnic; SYKT, Sanyang Xuedai; SMI, Shenmai Injection; Cts, Cryptotanshinone; RES, Resveratrol; HG, Higenamine; OIE, Oroxylum).

The mitogen-activated protein kinase (MAPK) pathway is a common signaling pathway that transmits extracellular signals to downstream effector molecules and is involved in physiological processes such as cell proliferation, differentiation, and apoptosis (101), which consists of three branches: MAPK, extracellular signal-regulated kinases (ERKs) and c-Jun-terminal kinases (JNKs) (102). The p53 pathway is another critical pro-apoptotic pathway. P53 upregulates the pro-apoptosis B-cell lymphoma-2(Bcl-2) associated X(Bax) protein, downregulates the anti-apoptosis protein Bcl-2, and activates the transcription of Fas and other death receptor genes (103). Additionally, IR-induced DNA damage initiates apoptosis through a p53-dependent mechanism that activates downstream caspases (104). STS can disrupt the p53 pathway in CFs by decreasing the phosphorylation levels of p38, caspases 3 expression, and increasing the levels of Bax and phosphorylated ERK1/2 (49). DBD can reduce Fas ligand (Fasl) and TNF-α expression, block apoptotic signaling pathways, and attenuate radiological myocardial injury in CMs (105) (Figure 4).

#### Anthracycline-based chemotherapy-induced apoptosis

DOX activates endogenous pathways and exogenous pathways of apoptosis (60). DOX downregulates the Akt pathway, induces caspases activity, and upregulates cell death receptors, all leading to CMs apoptosis (74). Blocking that impact, ginsenoside Rg1 increased Akt and ERK pathway phosphorylation, the ratio of Bcl-2 and Bax, and reduced Cytochrome C (Cyt-c) release from the mitochondria, thus disrupting DOX-induced CMs apoptosis (106). Additionally, ginsenoside Rb1 decreased caspase-3 and caspase-8 activity and blocked apoptosis in H9C2 cells (107). *Salvia miltiorrhiza* aqueous extract (SMAE) modulated ERK/p53/Bcl-xL/caspase-3 signaling pathway and improved mitochondrial dysfunction, significantly alleviating DOX-induced cardiomyopathy and apoptosis, and simultaneous administration of DOX and SMAE significantly inhibited the growth of breast cancer cells (108). SalB promoted Bcl-2 expression and attenuated DOX-induced apoptotic damage in cardiac tissue (109). Qishen Yiqi Dropping Pills (QYDP) increased vascular endothelial growth factor levels, myocardial microvascular density, and Bax expression, while it downregulated Bcl-2 and caspase 3 and attenuated MF in DOX-treated mice (110). AS-AM downregulated Bax, caspase 3, and caspase 12 and upregulated Bcl-2 expression. It also decreased apoptosis by inhibiting the intrinsic apoptotic pathway (111–113). Paeonol increased the viability and mitochondrial membrane potential (MMP) of DOX-induced CMs, upregulated the expression of Bcl-2 and mitochondrial Cyt c, downregulated the expression of Bax, caspase-3, and cytoplasmic-Cytc, and reduced apoptosis and ROS (114).

Members of the MAPK superfamily and PI3K are specifically involved in the induction of apoptosis and impairment of contractile function (102, 115). *Panax ginseng* glycoprotein protected against myocardial injury by inhibiting CMs apoptosis by upregulating the MAPK pathway (116). Diethyl blechnic (DB) activated the JNK1/2 pathway to protect CMs from cytotoxicity (117). Sanyang Xuedai (SYKT) has antioxidant properties and attenuates cardiotoxicity by inhibiting p53 and MAPK-induced apoptosis (118, 119). In a network pharmacology study, SMI increased PI3KCA and AKT1 expression, thus preventing CMs apoptosis (120). SMI reduced DOX-induced Bax/Bcl-2 and Caspase-3 levels and increased PI3K, p-Akt, and phosphorylated glycogen synthase kinase 3 beta (p-GSK-3β) levels in C57BL/6 mice. Similarly, cryptotanshinone (Cts) attenuated apoptosis *via* the Akt-GSK-3β-mPTP pathway (121).

AMP-activated protein kinase (AMPK) is at the center of DOX-induced cardiotoxicity. DOX has an inhibitory effect on cardiac AMPK, which increases cardiotoxicity (122). SMI increased AMPK phosphorylation levels, preventing DOX-induced excessive mitochondrial ROS generation, decreasing mitochondrial membrane potential, and reducing DOX-injured H9C2 cells from apoptosis (123). RES and matrine attenuated CMs apoptosis *via* the AMPK pathway (124, 125). Higenamine, the main active component of the TCM Wu-Tou, also attenuates DOX-induced cardiac remodeling and myocyte apoptosis by suppressing AMPK activation (126) (Figure 4).

#### Non-anthracycline-based chemotherapy-induced apoptosis

*Oroxylum indicum* extract (OIE) significantly reduces caspase-3 and protease activity in the hearts of DOX- and CP-treated C57BL/6 J mice (127). Maltol (produced by heating *Panax ginseng*) enhanced PI3K/Akt expression levels and reduced CP-induced apoptosis in H9C2 cardiomyocytes during cisplatin treatment (128) (Figure 4).

Radiotherapy induced the activation of MAPK and P53-dependent apoptotic pathways, and STS and DBD inhibited apoptosis through P53 and Fas/TNF-α pathways; studies on the inhibition of chemotherapy-induced cardiomyocyte apoptosis by TCM mainly focused on apoptosis-related genes such as Bax, Bcl-2, and MAPK and AMPK pathways. Other apoptotic pathways, such as the mitochondrial apoptosis pathway, have not been as thoroughly investigated, which may suggest a direction for future research.

### Autophagy

Autophagy is a major regulator of homeostasis and heart function (129, 130). DOX regulates upstream regulatory processes of autophagy, such as mTOR and AMPK, and PI3K is also hyperactivated in a rat model of DOX cardiotoxicity (131). PI3K CI activates AKT, and activated AKT1 further activates mTORCl; mTORC2 is a bidirectional regulator of autophagy. mTORC2 indirectly inhibits autophagy through the AKT1/FOXO3a axis, and activated AKT1 leads to translocation of FOXO3 from the nucleus, thereby inhibiting autophagy-associated genes microtubule-associated protein light chain-3 (LC3) transcription (132). Activated AMPK directly promotes autophagy by phosphorylating mTORC1, ULK1, and autophagy-associated proteins in the PIK3C3/VPS34 complex (133).

TCMs can disrupt the ability of DOX to induce autophagy. DBD activated the PI3K pathway to inhibit CM autophagy in mice (134). QSHWC consists of 19 TCMs. Network pharmacological studies revealed that QSHWC contains 35 major active ingredients that can reduce the cardiotoxicity of anthracyclines by regulating PI3K/Akt, MAPK, FOXO, and other signaling pathways to regulate cellular autophagy and reduce the cardiotoxicity of anthracyclines (135), QSHWC downregulated pirarubicin-induced LC3, and played a cardioprotective role by inducing PI3K, AKT, and mTOR phosphorylation and pathway activation (136). Qiliqiangxin (QL), a compound used in TCM, protected against cardiotoxicity by deactivating the PI3K/AKT/mTOR pathway to inhibit autophagy (137). The protective effect of RES against DOX cardiotoxicity is largely dependent on its ability to regulate autophagy through the AMPK/mTOR/Ulk1 signaling pathway (138).

**Figure 5:**
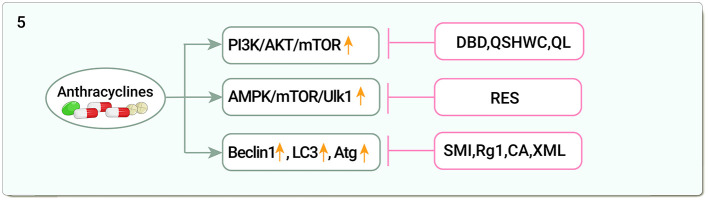
Traditional Chinese medicine alleviates cardiotoxicity associated with chemoradiotherapy by regulating autophagy (PI3K/AKT/mTOR, phosphatidylinositol 3-kinase/serine-threonine protein kinasem/Mechanistic Target Of Rapamycin; AMPK/mTOR/Ulk1, Adenosine monophosphate-activated protein kinase/Mechanistic Target Of Rapamycin /Unc-51-like autophagy activated kinase; LC3, Light chain-3; Atg, Autophagy-related genes; DBD, Danggui Buxue decoction; QSHWC, Qishen Huanwu Capsule; QL, Qiliqiangxin; RES, Resveratrol; SMI, Shenmai Injection; Rg1, Ginsenoside Rg1; CA, Calycosin; XML, Xinmailong injection).

Beclin 1 was identified as a Bcl-2-interacting protein that is essential for autophagy (139). DOX-induced mitochondrial autophagy was evidenced by increased Beclin 1, LC3, decreased p62, and co-localization of LC3 in mitochondria (140). LC3 is associated with the development and maturation of autophagic vesicles (141). SMI inhibited excessive myocardial autophagy by downregulating Beclin1 expression and attenuated DOX-induced myocardial injury (142), in an *in vitro* model of DOX-induced cardiotoxicity and also attenuated myocardial cell damage by deactivating the JNK signaling pathway and blocking autophagy formation (143). Ginsenoside Rg1 reduced DOX-induced LC3, autophagy-related genes (Atg) 5, and Beclin 1 expression and improved cardiac insufficiency (144). CA exerted cardioprotective effects through Atg7 by promoting autophagic vesicle formation in a DOX-induced zebrafish embryonic heart injury model (145). XML reduced Beclin 1 and Atg7 accumulation, upregulated protein kinase B (PKB)/Akt, PI3K, and Bcl-2 levels, and inhibited autophagy to alleviate cardiomyopathy (146) (Figure 5).

DOX induces autophagy through activation of PI3K/AKT/mTOR, and AMPK/mTOR key pathways as well as autophagy-related genes such as Beclin 1, LC3, and Atg. TCM regulates autophagy through these pathways and alleviates DOX-induced cardiotoxicity. In addition to these mechanisms, whether non-coding RNAs regulate autophagy, epigenetics of autophagy, protein modification and autophagy activation, and other important transcription factors such as FOXO, E2F, and TFEB, which are involved in the regulation of autophagy, can become new targets of anti-DOX cardiotoxicity in TCM, needs to be confirmed by a large number of studies, which also provides us with a direction for future research.

### Endoplasmic reticulum stress

**Figure 6:**
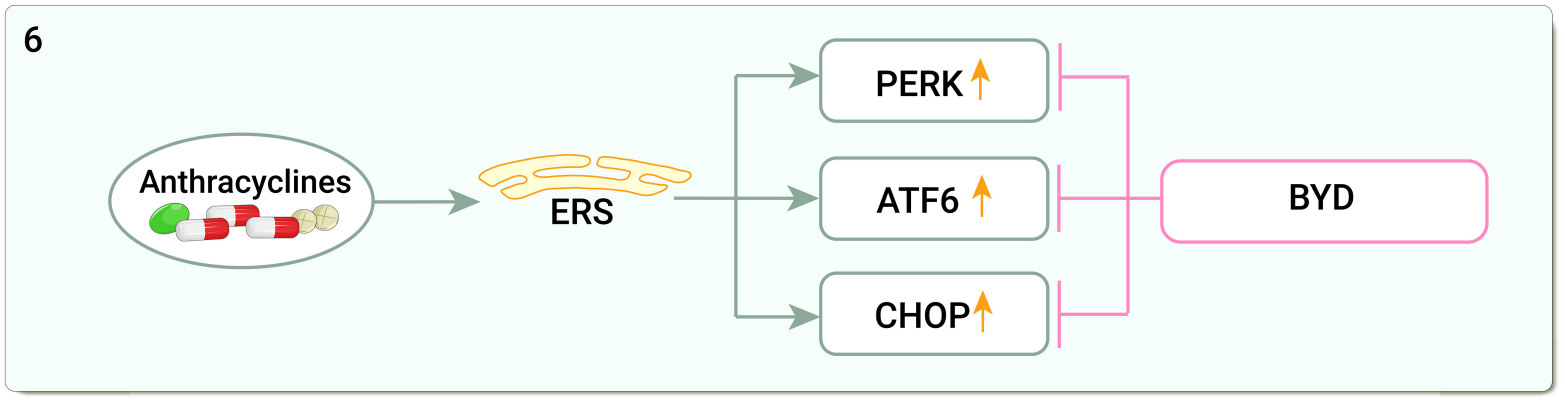
Traditional Chinese medicine alleviates cardiotoxicity associated with chemoradiotherapy by inhibiting endoplasmic reticulum stress (ERS, Endoplasmic reticulum stress; PERK, The stress protein kinase R-like endoplasmic reticulum kinase; ATF6, Activating transcription factor 6; CHOP:C/EBP homologous protein; BYD, Baoyuan decoction).

DOX can cause marked endoplasmic reticulum (ER) expansion in the human heart (147), upregulating the stress protein kinase R-like endoplasmic reticulum kinase (PERK), C/EBP homologous protein (CHOP), and activating transcription factor 6 (ATF6) in cardiac tissue (148). Baoyuan decoction reduced glucose regulated protein78 (GRP78), PERK, eukaryotic translation initiation factor 2-alpha (eIF2α), and CHOP protein and mRNA expression and ameliorated DOX-induced myocardial injury by inhibiting CMs apoptosis by downregulating the endoplasmic reticulum stress (ERS) apoptotic pathway (149) (Figure 6).

Studies on the mitigation of cardiotoxicity by TCM through ERS are limited and restricted to three pathways of ERS itself. In fact, many cellular processes including inflammation, apoptosis, and autophagy are regulated by the ERS pathway, and the importance of ER and its signaling pathways in inflammation, apoptosis, and autophagy in DOX-induced cardiotoxicity suggests that it may be a key factor in reducing DOX-induced cardiotoxicity (148).

### Myocardial energy metabolism

**Figure 7:**
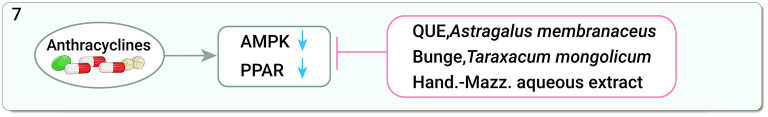
Traditional Chinese medicine alleviates cardiotoxicity associated with chemoradiotherapy by regulating myocardial energy metabolism (AMPK, adenosine monophosphate-activated protein kinase; PPAR, peroxisome proliferator-activated receptors; QUE, Quercetin).

DOX impairs most of the processes of myocardial energy metabolism through oxidative phosphorylation, the mitochondrial respiratory chain, and the AMPK signaling pathway, leading to significant downregulation of AMPKα2, peroxisome proliferator-activated receptors α (PPARα), and the peroxisome proliferator-activated receptor γ-coactivator 1α (PGC-1α) expression and affecting cardiac function (150, 151). QUE regulates the AMPK signaling pathway by promoting AMPKα2, PPARα, and PGC-1α expression to improve myocardial energy metabolism and prevent DOX-induced cardiac damage in rats (152). *Astragalus membranaceus* Bunge promotes fatty acid metabolism and activates PPARγ in DOX-induced heart failure in mice to maintain fatty acid homeostasis in H9C2 cells, thereby alleviating myocardial injury (153). *Taraxacum mongolicum* Hand.-Mazz. aqueous extract can activate P-glycoprotein in the cardiac tissue of triple-negative breast cancer patients and ameliorate DOX-induced cardiotoxicity (154) (Figure 7).

Although the oxidation of mitochondrial fatty acids and carbohydrates is the main source of ATP production in the heart, the oxidation of other energy substrates, such as ketones and branched-chain amino acids, also contributes to energy production (155), and the use of TCM to improve myocardial energy metabolism to alleviate cardiotoxicity may be a promising research direction.

## Discussion

The field of oncological cardiology formed due to the realization that cancer treatment-related cardiovascular disease is a major challenge for both cardiologists and oncologists (156). Aging populations and advances in diagnosis and treatment have improved survival rates for patients with cancer (157) but have also increased the incidence of cancer treatment-related cardiotoxicity (158). Cardiotoxicity is the result of a combination of mechanisms, and there are no effective western drugs that can reverse this damage. TCMs are unique in the treatment of chemoradiotherapy-related cardiotoxicity because of their single-target superposition, multi-target synergy, toxicity dispersion effects in many potent forms, and their ability to weaken their own toxicity (159). This paper summarizes six important mechanisms of TCM in the treatment of chemoradiotherapy-related cardiotoxicity: anti-OS and inflammation, regulation of apoptosis and autophagy, alleviation of ERS, and improvement of myocardial energy metabolism. The molecules and pathways involved include ROS, Noxs, Nrf2, TGF-β/Smad, NF-κB, NLRP3, P53, PI3K/AKT, MAPK, AMPK, PI3K/AKT/mTOR, AMPK/mTOR/Ulk1, Beclin1, LC3, Atg, ERS Pathway, ATP, ADP and PPAR, showing that the broad role and good effect of TCM in the treatment of chemoradiotherapy-related cardiotoxicity.

Among the many anti-cardiotoxic TCMs summarized in this paper, some drugs have attracted our attention, including various extracts and active ingredients of *Salvia miltiorrhiza*: STS, Tan I, DSS, SalB, DHT, danshensu, Cts, DB, CDDP; *Astragalus membranaceus* Bunge and its extracts AS-IV and CA; active ingredients of *Panax ginseng*: Ginsenoside Rg1, Ginsenoside Rb1, *Panax ginseng* glycoproteins, and Maltol. Soup containing *Angelica sinensis* DBD and AS-AM. *Salvia miltiorrhiza* is a well-known herb with a wide range of cardiovascular protective effects. Previous studies have shown that the lipophilic components (tanshinone I, tanshinone IIa, tanshinone IIb, cryptotanshinone, dihydrotanshinone, etc.) and the hydrophilic components (danshensu, salvianolic acid A and B, protocatechuic aldehyde, etc.) are involved in the cardioprotective effects of *Salvia miltiorrhiza* (160). Tanshinone IIA (Tan IIa) is a lipid-soluble compound isolated from the traditional Chinese medicine *Salvia miltiorrhiza* (161), and STS is a water-soluble derivative of Tan IIa (162), which can effectively inhibit the interaction between DNA and intracellular lipid peroxidation products (163) and can alleviate cardiotoxicity by anti-OS and reducing apoptosis. *Astragalus membranaceus* Bunge is the holy medicine of supplement Qi, which has the effect of supplement Qi, raising Yang, nourishing the Wei Qi, and fixing the surface. Eight key components in *Astragalus membranaceus* Bunge, including hederagenin, quercetin, calycosin, formononetin, jaranol, isorhamnetin, astragaloside III, and 9,10-dimethoxypterocarpan-3-O-β-D-glucoside, are involved in lipid metabolism, programmed cell death, fatty acid metabolism, which produce the ability to regulate the body's immune function, strengthen the heart, protect CM, improve substance metabolism (164), AS-IV is a cyclic aromatic triterpene glycoside compound, which is one of the main active components of *Astragalus membranaceus* Bunge and has good antioxidant activity (165), and can alleviate cardiotoxicity by anti-OS and improving myocardial energy metabolism. *Panax ginseng* is a widely used herb in the world, containing more polysaccharides and amino acids, with better protective effects against cardiovascular diseases, neurological diseases, cancer, and diabetes (166). Ginsenosides are the main active components of ginseng, which can reduce cardiotoxicity through anti-OS, reduce apoptosis and regulate autophagy. *Angelica sinensis* is one of the most popular traditional TCM, which has long been used as a blood tonic and blood activator, pain reliever, laxative, and treatment of female menstrual disorders and amenorrhea. It contains polysaccharides, ligustrolactone, ferulic acid, and other bioactive components, with antioxidant, anti-inflammatory, anti-fibrotic, and cardiocerebrovascular protective effects (167). DBD is a classical formula in TCM to supplement Qi and replenish blood. It is composed of *Astragalus membranaceus* Bunge and *Angelica sinensis* in a 5:1 ratio, and DBD and its extract AS-AM alleviate cardiotoxicity through various pathways such as anti-OS, inhibition of apoptosis and TGF-β overexpression, and reduction of autophagy. In conclusion, *Salvia miltiorrhiza, Astragalus membranaceus* Bunge*, Panax ginseng*, and *Angelica sinensis* are important TCMs against cardiotoxicity and should be given more attention.

Although significant progress has been made in exploring the molecular mechanisms of TCM against chemoradiotherapy-related cardiotoxicity, research on TCM against cardiotoxicity is still facing some problems and shortcomings. (1) The studies on the anti- chemoradiotherapy-related cardiotoxicity of TCM are limited to OS, inflammation, apoptosis, autophagy, ERS, and myocardial energy metabolism, but other important mechanisms such as Top2β have not been addressed, and many investigations into TCM have only studied one of these mechanisms, which is not conducive to our comprehensive understanding of the mechanisms of anti-cardiotoxicity of TCM. (2) Most of the studies were focus on chemotherapy-induced cardiotoxicity (particularly anthracyclines), and fewer studies were done on radiotherapy and non-anthracyclines. Among the six mechanisms summarized in this paper, only two mechanisms of OS and apoptosis were involved in radiotherapy, and even fewer in non-anthracyclines. However, the cardiotoxicity caused by radiotherapy and non-anthracycline drugs is worthy of attention, such as myocarditis caused by immune checkpoint inhibitors and the decrease of LVEF caused by arsenic trioxide, which may be alleviated by TCM, and this also broadens the idea of research on the effects of TCM on anti-radiotherapy cardiotoxicity. (3) Most of the studies on anti-cardiotoxicity of TCM are limited to the cellular level and animal trials, and till date, only a few clinical control studies have been conducted, with small sample size and irregular design, and the reproducibility of many therapies and prescriptions is poor. (4) The specific medicinal components and active parts of some herbal monomers, extracts, and compounds are not clear, and their targets are unknown, so high-performance liquid chromatography and mass spectrometry may be needed to identify the drug components and lay a clearer material basis for pharmacological research. (5) There is a lack of knowledge about the safety of TCM and their interactions with western drugs, which also limits the applications of TCM. (6) TCM emphasizes both a holistic view of the body and evidence-based treatment; patients with similar symptoms may be treated with different drugs because they suffer from different conditions, which requires the establishment of a systematic set of diagnostic and therapeutic criteria for better clinical treatment and research. (7) This paper only includes the studies on the cardiotoxicity caused by radiotherapy, anthracycline chemotherapy and two non-anthracycline chemotherapy drugs, CP and cyc, but not the studies on the cardiotoxicity caused by other drugs, such as immune checkpoint inhibitors (ICIs), arsenic trioxide and targeted chemotherapy drugs, which may make this study less comprehensive.

In addition to cardiotoxicity caused by radiotherapy and classical chemotherapeutic agents, ICIs, arsenic trioxide, and other antineoplastic agents may cause cardiotoxicity. ICIs, a unique antibody-based therapeutic strategy that has revolutionized the treatment landscape for solid and hematologic cancers, has been shown in a growing number of preclinical studies to trigger myocardial inflammation, and the incidence of cardiotoxicity in ICIs therapy may be underestimated (168). Cardiac immune-associated adverse events are rare but potentially fatal complications of immunotherapy with various potential risk factors, such as combinations of different ICIs (169). No studies related to the cardiotoxicity of TCM on ICIs have been retrieved, but this may be a neglected area of study. For TCM treatment of arsenic trioxide-induced cardiotoxicity, relatively few studies were found. One such studies indicated that crocin ameliorates arsenic trioxide-induced cardiotoxicity by reducing OS, inflammation, and apoptosis (170, 171), magnesium isoglycyrrhizinate attenuates *via* Nrf2 and TLR4/NF-κB signaling pathways arsenic trioxide-induced cardiotoxicity (172). In addition, microRNAs and non-coding RNAs are also involved in the pathogenesis of radiotherapy-associated cardiotoxicity (173–175), mitochondrial fusion (176), and cellular scorching (177). It is also a hot topic of current research that TCM can exert anti-cardiotoxic effects through these pathways, and we look forward to seeing more of these findings.

## Conclusion

Cardiotoxicity development is the result of a combination of mechanisms. In recent years, the benefits of TCM in chemoradiotherapy-related cardiotoxicity have become evident. Herbal monomers, such as AS-IV and STS, herbal decoction, such as DBD and SXT, or compound preparations, such as SMI and QSHWC, can protect CMs through antioxidation, anti-inflammation, regulating autophagy and apoptosis, inhibiting ERS, and improving myocardial energy metabolism, and play a role in reducing anti-radiotherapy-related cardiotoxicity. Moreover, TCM is a promising drug for treating chemoradiotherapy-related cardiotoxicity, both guided by TCM theory and supported by modern research. However, our conclusions are based on numerous basic, smaller experiments and lack the results of large-scale clinical trials. We look forward to more relevant randomized controlled trials to show the benefits of TCM on chemoradiotherapy-related cardiotoxicity.

## Author contributions

X-FL, R-QW, Y-DL, KL, and X-KZ designed the study. X-FL, XG, XW, and X-DZ acquired and researched the data for the article. C-LP, C-ZR, and Q-LC discussed its content. X-FL wrote the manuscript. C-LP, W-JL, and T-YB revised the manuscript. All authors read and approved the final manuscript.

## Funding

This work was partly supported by the National Natural Science Foundation of China (No. 81873132), the Science and Technology Innovation Project of Gansu Provincial Education Department (No. 2021jyjbgs-03), and the Gansu Provincial Scientific Research Project on Prevention and Treatment of Major Diseases in Traditional Chinese Medicine (No. GZKZD-2018-02).

## Conflict of interest

The authors declare that the research was conducted in the absence of any commercial or financial relationships that could be construed as a potential conflict of interest.

## Publisher's note

All claims expressed in this article are solely those of the authors and do not necessarily represent those of their affiliated organizations, or those of the publisher, the editors and the reviewers. Any product that may be evaluated in this article, or claim that may be made by its manufacturer, is not guaranteed or endorsed by the publisher.
